# Practicing evidence based medicine at the bedside: a randomized controlled pilot study in undergraduate medical students assessing the practicality of tablets, smartphones, and computers in clinical life

**DOI:** 10.1186/s12911-014-0113-7

**Published:** 2014-12-05

**Authors:** Hendrik Friederichs, Bernhard Marschall, Anne Weissenstein

**Affiliations:** University of Muenster, Studienhospital der Medizinischen Fakultät Münster, Malmedyweg 17-19, 48149 Muenster, Germany; Institute of Medical Education – IfAS, University of Muenster, Albert – Schweitzer – Strasse 21, 48149 Muenster, Germany

**Keywords:** Usability, Mobile device, Smartphone, Tablet computer, Evidence based medicine

## Abstract

**Background:**

Practicing evidence-based medicine is an important aspect of providing good medical care. Accessing external information through literature searches on computer-based systems can effectively achieve integration in clinical care. We conducted a pilot study using smartphones, tablets, and stationary computers as search devices at the bedside. The objective was to determine possible differences between the various devices and assess students’ internet use habits.

**Methods:**

In a randomized controlled pilot study, 120 students were divided in three groups. One control group solved clinical problems on a computer and two intervention groups used mobile devices at the bedside. In a questionnaire, students were asked to report their internet use habits as well as their satisfaction with their respective search tool using a 5-point Likert scale.

**Results:**

Of 120 surveys, 94 (78.3%) complete data sets were analyzed. The mobility of the tablet (3.90) and the smartphone (4.39) was seen as a significant advantage over the computer (2.38, *p* < .001). However, for performing an effective literature search at the bedside, the computer (3.22) was rated superior to both tablet computers (2.13) and smartphones (1.68). No significant differences were detected between tablets and smartphones except satisfaction with screen size (tablet 4.10, smartphone 2.00, *p* < .001).

**Conclusions:**

Using a mobile device at the bedside to perform an extensive search is not suitable for students who prefer using computers. However, mobility is regarded as a substantial advantage, and therefore future applications might facilitate quick and simple searches at the bedside.

**Electronic supplementary material:**

The online version of this article (doi:10.1186/s12911-014-0113-7) contains supplementary material, which is available to authorized users.

## Background

“Evidence-based medicine is the conscientious, explicit, and judicious use of current best evidence in making decisions about the care of individual patients” [1]. This requires the integration of individual clinical expertise with the best available external clinical evidence from systematic research. According to Sackett et al., neither individual clinical expertise nor the best available external evidence alone are sufficient to be a good doctor—they have to be combined [1]. That is why physicians require both knowledge of practical skills and expertise in the best methods of gathering external evidence-based information. The implementation of evidence based medicine (EBM) is realized by reviewing all information in the international literature relevant to a particular patient before a diagnostic or therapeutic medical intervention. External evidence is examined for its general and special validity and is thereafter integrated in the medical decision making process [2].

Currently, the most effective way to obtain external evidence is a literature search using computer-based systems [2]. Electronic databases have the advantage of being easy accessible. Accessibility is an important factor determining that information-seeking is likely to occur and likely to be successful [3]. Of all databases, Medline is the world’s largest and most important medical literature database. It is the electronic equivalent of Index Medicus, Index to Dental Literature, and International Nursing Index. It can be accessed from numerous online services by using an appropriately equipped computer (PC).

EBM’s easy accessibility and rapid rate of search is important because physicians rarely choose to seek information during a patient encounter [4]. According to Ely et al., physicians search for answers in only 55% of cases in which questions arise [5]. Reasons include lack of time, the large amount of material, the belief that there is likely to be no answer, forgetfulness, and lack of urgency [3,6,7]. Searching for external evidence is important because most of the information physicians use when seeing patients is obtained from memory and some is out of date or wrong [8]. A study by Gonzalez-Gonzalez et al. has shown that when physicians chose to search for answers during consultation, they were successful 100% of the time [9]. In contrast, the success rate for searches performed after a consultation was 75% [9]. An immediately accessible information search using a mobile device would considerably increase the frequency of searching for answers, [10,11] because when evidence is not readily available, physicians rarely search for it. If it were possible for clinicians to search for evidence at the bedside, this would have many advantages. If each clinician had an appropriate device to search for external information near the patient, the chances are higher that he or she might use it and that it might affect diagnostic and/or treatment decisions.

While there are plenty of mobile devices available to fulfill this need, no studies exist comparing the usability of smartphones, tablets and stationary PCs at the bedside. For this reason, the medical faculty in Muenster, Germany conducted a pilot study with the following aims:

1. To evaluate the internet use habits of students.
2. To reveal possible differences between the various tools and to assess whether the use of tablets and smartphones at the bedside or the classic use of PCs in doctors’ rooms results in an improved self-assessment of the students’ professional knowledge and skills.
3. To determine whether this new curriculum in the field of EBM is being accepted by students.

## Methods

### Study design and participants

In a randomized controlled pilot study, 120 third-year students (first clinical semester) were divided into three groups. One group served as controls and solved clinical problems on a PC that was located in a doctor’s room on the ward, as is so often the clinical reality. The second group was an intervention group that represented the growing use of smartphones. They were given iPods, which are about the same size as the iPhone but that lack the ability to make phone calls. The third group was equipped with tablet computers (iPads). Each group was further divided into sub-groups with a maximum of six students because of space limitations in the training rooms. The effectiveness of the devices was assessed with a questionnaire. Informed consent was provided by all participants before the study and the Ethics Committee of the Chamber of Physicians Westfalen-Lippe and the Medical Faculty of the Westfalian Wilhelms University Muenster waived requirements for an ethical approval procedure.

### Intervention

Before the study, a training period was conducted allowing the students to practice finding literature to answer a clinical question. In a case study, a patient suffering from a common cold was presented in an outpatient environment by video. The patient asked the doctor about various treatment methods and their effectiveness. All three groups performed a literature search to get accustomed to their respective search device. A search algorithm corresponding to the recommendations given by the PubMed website [12] to help students find relevant literature was provided. Students searched using the online platform “Unbound Medline” because it provides the same interface for all mobile devices and therefore search conditions were standardized. In addition, the software proposes MeSH terms when entering medical terminology which correspond to the search with the MeSH database.

After this training period, each group visited three simulated patients with different illnesses in a hospital setting. It was their task to perform a search using the now-familiar algorithm on a question resulting from the examination. This search was performed, depending on the device, either directly at the bedside or in a doctors’ room. Two of the six students forming a group visited the patient with a student tutor, while the other four observed them from behind mirrored glass. Two of the observing students evaluated the doctor–patient communication during the scene. The other two observing students performed the same search algorithm under the supervision of a student tutor. The aim for each topic was to find the corresponding Cochrane review, which was subsequently given to the group as a handout.

### Questionnaire

In a questionnaire (see Additional file 1), students were asked to provide a self-assessment concerning various items on a 5-point Likert scale. The scale ranged from values of one (“strongly disagree”) to five (“strongly agree”). An answer of five represents the highest level of agreement. Preliminary questions established baseline data. Students were asked how frequently they used a PC or smartphone with internet access, how frequently they used the internet, and whether they used it for scientific literature search. Subsequently, students were asked to provide a self-assessment of their skills using their respective search device.

### Data analysis

Data were analyzed with IBM Statistics SPSS 19. Descriptive means and standard deviations were calculated. We used analysis of variance (ANOVA) and chi-square tests to evaluate whether students’ baseline characteristics and students’ internet access, internet use, and frequency of literature search were balanced between groups. Significance was set at a = 0.05. We evaluated ordinal scales parametrically following the guidance of Norman (2010) [13].

## Results

### Response rate

Of 120 distributed questionnaires, 118 could be retrieved after the course. After evaluating all data, 108 questionnaires could be assigned specifically to one person . Of these questionnaires, those with a completion rate of more than 90% were regarded as appropriate for analysis, resulting in 94 (78.3%) complete data sets for analysis.

### Participants’ characteristics and baseline data

The remaining 94 questionnaires were sorted into three groups. We obtained 31 questionnaires in the tablet group, 31 surveys in the smartphone group, and 32 questionnaires in the PC group. The baseline characteristics (age and gender) of students as well as students’ habits regarding internet access, internet use, and literature search frequency did not differ significantly between the three randomized groups.

Students’ mean age was 22.6 years and 61.7% were female. All students possess and use a PC with internet access, 94.6% of which were laptops/notebooks. One quarter of all students (25.5%) have mobile internet access, 20 students (19.2%) have a smartphone with internet access, two (.2%) have an iPod touch, and four students (.4%) have an iPad. Nearly all students (96.8%) use the internet at least daily, and more than half (58.5%) perform a literature search at minimum weekly on medical issues (whether in books, journals, or the internet). A majority of students (55.3%) use the internet to do their searches. However, mobile devices are rarely used for literature searches (16.0%). Only 3.2% of all students perform a weekly literature search with PubMed/Medline, while 12.8% do so monthly.

### Pilot study

The results of the questionnaires can be seen in Table 1. To assess emotional involvement, the participants were asked about their perception of fun during the course. In this regard, there were no significant differences between groups. However, the students that were randomly assigned to the PC group reported the highest degree of agreement that the practical day was worthwhile (3.22), in contrast to smartphone (2.87) and tablet groups (2.61). The difference between the tablet and PC groups was significant (*p* = .035). All groups reported equal levels of satisfaction when asked whether they had sufficient technical skills for the bedside literature search. No difference between was found concerning students’ degree of confidence in performing a literature search at the bedside. The students in the PC group were significantly more motivated to deepen their knowledge (3.25) than the tablet group (2.58; *p* = .016).

**Table 1:** **Questionnaire results of each of the three groups**

**Questionnaire**	**Tablet group (sd)**	**Smartphone (sd)**	**Computer group (sd)**
The practical day of evidence based medicine at the bedside was fun	2.84 (1.10)	3.0 (1.34)	2.97 (1.26)
The practical day of evidence based medicine at the bedside was worthwhile	2.61 (1.15)	2.87 (1.36)	3.22 (1.10)
I have sufficient technical skills in the literature search at the bedside	2.71 (.97)	2.84 (.90)	2.69 (.78)
I feel confident enough in the literature search at the bedside	2.58 (.88)	2.39 (.88)	2.56 (.84)
I am motivated to further work on the topic of literature at the bedside	2.58 (1.15)	2.87 (1.26)	3.25 (.98)
The literature search performed at the bedside was easy	2.29 (1.01)	1.77 (.99)	2.78 (1.21)
The literature search at the bedside was effective	2.13 (1.23)	1.68 (.87)	3.22 (1.31)
I was satisfied with my search instrument	3.35 (1.33)	2.74 (1.40)	3.63 (1.41)
I was satisfied with the screen size of my search instrument	4.10 (1.14)	2.00 (1.34)	4.06 (1.30)
I was satisfied with the mobility of my search instrument	3.90 (.94)	4.39 (1.09)	2.38 (1.34)
I was satisfied with the handling of my search instrument	3.52 (1.21)	3.19 (1.25)	3.88 (1.41)
I will try the literature search at the bedside in my next internship	2.16 (1.16)	1.87 (1.09)	2.88 (1.21)

Answers were provided using a 5-point Likert scale, where 1 = “strongly disagree” and 5 = “strongly agree”.

The PC group found searching easier (2.78) than those students who used a smartphone (1.77), and this difference was highly significant (*P* < .001). Furthermore, the PC group found the search more effective (3.22) than students using the smartphone (1.68, *p* < .001) or tablet (2.13, *p* < .001). These differences were highly significant. Students who used the PC reported being most content with their search device (3.63), in comparison to the smartphone group (2.74) and the tablet group (3.35). Students in the PC group were as satisfied with the size of their screen (4.06) as the tablet group (4.10), while the smartphone group was less satisfied (2.00). Satisfaction concerning mobility was especially high in the smartphone group (4.39) and the tablet group (3.90), while the PC group only reported a satisfaction level of 2.38, which was significantly lower than either portable group (*p* < .001).

No significant differences in satisfaction were found with search instrument handling between tablets (3.53) and smartphones (3.19). The PC group was the most satisfied with handling (3.88), which was significantly greater than the mobile groups (*p* = .044). Of all students, those who used the PC (2.88) were most eager to try a literature search during their next internship compared with the tablet group (2.16, *p* < .001) and the smartphone group (1.87, *p* < .001).

## Discussion

In our pilot study, we found that the PC retains its superior position when compared with mobile devices in performing an effective literature search, and PC use led to significantly greater determination to practice EBM in the next internship. We found no significant differences between tablets and smartphones beside satisfaction with screen size. However, the mobility of the tablet and smartphone is seen as an advantage over the PC. The proposed MeSH terms provided by the software when entering medical terminology were a big help for students, especially for non-native English speakers. A correspondence between the search and the MeSH database could be achieved in seconds, which allows the use of mobile devices in principle. When a MeSH term was mistakenly used in the search and the search had to be started from the beginning, correcting the results did not always work reliably with the newly selected MeSH term.

Despite the increasing trend in the use of technical devices by physicians, with adoption rates ranging from 45% to 85% in recent years [14,15], there are few studies evaluating and comparing the usability of smartphones, tablets, and stationary computers. Several studies have explored the uses and benefits of handheld computers alone [16-19]. However, the majority of research in this area is descriptive rather than quantitative. In only one study, the author quantitatively compared the usability of the iPod touch and the palm Tungsten C [13]. The results of their study indicated that perceived ease of use is the most important factor affecting physicians’ intentions to use handheld computers. This agrees with the findings of our study, in which perceived ease of use was significantly higher in the PC group. However, the key advantage of smartphones and tablets lies in their mobility and thus the possibility to use them at the bedside. They have been shown to improve point of care, medical decision making, clinical communication, patient education, and overall coordination of patient care [20]. Despite the numerous advantages of mobile devices at the bedside, our students were not satisfied with their portable tools. One reason for participants’ dissatisfaction with smartphones and tablets may be the screen size. Especially when entering MeSH terms to perform a literature search, the dimension of the display plays a very important role. Another explanation may be that the mobile versions of databases are not yet optimized for doing an extensive search.

There are several limitations to our study. First, we did not exclude participants with prior experience using smartphones and tablet computers, and this may have led to bias. Second, students only evaluated the devices for a short period of time. Consequently, our results may not reflect usability in day-to-day clinical life and their generalizability may be limited. Third, participants only used the search platform Unbound Medline. The usability of the devices might differ if other platforms were tested. Important databases including the Cochrane Library and the ACP Journal Club were not used in our study. A further aspect is the high cognitive load resulting from the high amount of results when using Unbound Medline. It can be argued that other sources, such as UpToDate or BMJ Clinical Evidence are designed to present an integrated knowledge, however, this can be subject to biases in interpretation which is the reason why we have preferred to work with the original data, especially with undergraduates. Further, with the aim of reducing the cognitive load we have focused on meta-analyses in our search.

## Conclusion

Finally, it can be concluded that using a mobile device at the bedside to perform an extensive literature search is not yet suitable for students, who prefer doing this on the familiar screen of a PC. Nevertheless, the use of mobile devices at the bedside is being accepted by students, and all groups reported that they felt they had sufficient technical skills and confidence to perform a literature search at the bedside. Further research is needed to assess whether other ways of providing EBM at the bedside such as mobile PCs on ward trolleys would compensate for the deficits of stationary PCs.

## Additional file

Additional file 1
**Evaluation for the Curriculum “Individual Knowledge Management”.**
