# Supplementary material for: Practicing evidence based medicine at the bedside: a randomized controlled pilot study in undergraduate medical students assessing the practicality of tablets, smartphones, and computers in clinical life
Source: BMC Med Inform Decis Mak. 2014 Dec 5;14:113. doi: 10.1186/s12911-014-0113-7 (PMC4262131; doi:10.1186/s12911-014-0113-7)
Supplement: Additional file 1 — Evaluation for the Curriculum “Individual Knowledge Management”. [file 12911_2014_113_MOESM1_ESM.docx]

Evaluation for the Curriculum „Individual Knowledge Management“:

How old are you? _________ years

Gender? ❒ male ❒ female

Do you possess and use a personal computer (pc) with internet access?

❒ yes, a stationary pc ❒ yes, a laptop/notebook (Multiple answers possible)

Do you possess and use a mobile internet access via **cell phone/smartphone or iPod touch/iPad**?

❒ yes, a cell phone ❒ yes, a smartphone ❒ yes, an iPod touch ❒ yes, an iPad ❒ no

Type ❒ iPhone

❒ other (Multiple answers possible)

How often do you use the internet?

❒ hourly ❒ daily ❒ weekly ❒ monthly ❒ not at all

How often do you generally do a **literature search** (books, journals, internet, …) on **medical topics?**

❒ hourly ❒ daily ❒ weekly ❒ monthly ❒ not at all

How often do you perform a literature search on medical topics **on the internet?**

❒ hourly ❒ daily ❒ weekly ❒ monthly ❒ not at all

How often do you perform a literature search on medical topics via **mobile internet access with a cell phone/smartphone or iPod touch/iPad** **?**

❒ hourly ❒ daily ❒ weekly ❒ monthly ❒ not at all

How often do you perform a literature search **on PubMed/Medline** on medical topics?

❒ hourly ❒ daily ❒ weekly ❒ monthly ❒ not at all

**→**

|  | strongly agree | agree | partly agree | disagree | strongly disagree |
| --- | --- | --- | --- | --- | --- |
|  | | | | | |
| The practical day of evidence based medicine at the bedside was fun | 🔾 | 🔾 | 🔾 | 🔾 | 🔾 |
| The practical day of evidence based medicine at the bedside was worthwhile | 🔾 | 🔾 | 🔾 | 🔾 | 🔾 |
| I have sufficient technical skills in the literature search at the bedside | 🔾 | 🔾 | 🔾 | 🔾 | 🔾 |
|  | | | | | |
| I feel confident enough in the literature search at the bedside | 🔾 | 🔾 | 🔾 | 🔾 | 🔾 |
| I am motivated to further work on the topic of literature search at the bedside | 🔾 | 🔾 | 🔾 | 🔾 | 🔾 |
| The literature search performed at the bedside was easy | 🔾 | 🔾 | 🔾 | 🔾 | 🔾 |
| The literature search at the bedside was effective | 🔾 | 🔾 | 🔾 | 🔾 | 🔾 |
|  | | | | | |
| I was satisfied… | | | | | |
| … with my search instrument | 🔾 | 🔾 | 🔾 | 🔾 | 🔾 |
| … with the screen size of my search instrument | 🔾 | 🔾 | 🔾 | 🔾 | 🔾 |
| … with the mobility of my search instrument | 🔾 | 🔾 | 🔾 | 🔾 | 🔾 |
| … with the handling of my search instrument | 🔾 | 🔾 | 🔾 | 🔾 | 🔾 |
|  | | | | | |
| I will try the literature search at the bedside in my next internship | 🔾 | 🔾 | 🔾 | 🔾 | 🔾 |

Thank you for your cooperation!
